# Non-Invasive Biomarkers for Eosinophilic Esophagitis: Latest Developments and Future Directions

**DOI:** 10.3390/jcm14238576

**Published:** 2025-12-03

**Authors:** Serena Marulo, Alessandra Macrì, Peppino Mirabelli, Laura Aurino, Rossella Turco, Raduan Ahmed Franca, Paolo Quitadamo

**Affiliations:** 1 Clinical and Translational Research Unit, Santobono-Pausilipon Children’s Hospital, 80129 Naples, Italy; s.marulo@santobonopausilipon.it (S.M.); a.macri@santobonopausilipon.it (A.M.); l.aurino@santobonopausilipon.it (L.A.); 2 Pediatric Gastroenterology and Hepatology Unit, Santobono-Pausilipon Children’s Hospital, 80129 Naples, Italy; r.turco@santobonopausilipon.it (R.T.); p.quitadamo@santobonopausilipon.it (P.Q.); 3 Health Services Department, AORN Santobono Pausilipon, 80123 Napoli, Italy; ra.franca@santobonopausilipon.it

**Keywords:** non-invasive, biomarker, eosinophilic esophagitis, diagnosis

## Abstract

Eosinophilic esophagitis (EoE) is an emerging disease characterized by chronic inflammation of the esophagus. EoE is a multifactorial disorder, likely resulting from the combination of genetic predisposition, epithelial barrier dysfunction, environmental risk factors, and allergen sensitization, which lead to type 2 inflammation of the esophagus. The clinical manifestations are related to esophageal dysfunction and include dysphagia, food impaction, heartburn, regurgitation, and food refusal. These symptoms are sometimes superimposable and can often be confused with the symptoms of gastroesophageal reflux disease. To-date, EoE diagnosis relies on endoscopic examination and histological analysis of esophageal biopsies, with the diagnostic criterion defined as the presence of ≥15 eosinophils per high-power field (eos/HPF). As a result, both the diagnostic and the subsequent disease monitoring processes, including assessment of treatment, efficacy, and remission status, require repeated endoscopic procedures. These procedures are rather invasive for patients, particularly in the pediatric population, and impose a significant financial strain on healthcare systems. Therefore, in recent years, substantial efforts have been made to identify novel non-invasive or minimally invasive biomarkers. This review aims at synthesizing the current findings and at categorizing the most promising biomarkers according to the different biological sources to ultimately enable earlier detection, reduce patient discomfort, and guide personalized treatment strategies.

## 1. Introduction

First described in the 1970s [1,2], eosinophilic esophagitis (EoE) is now recognized as a chronic, immune-mediated inflammatory condition of the esophagus. It is characterized by a Th2-type immune response and infiltration of eosinophils into the esophageal mucosa, triggering localized inflammation and tissue remodeling [3,4].

The pathogenesis of EoE is likely multifactorial, involving genetic, environmental, and immunological factors. Antigenic stimuli primarily drive the disease, most commonly food allergens, but also potentially environmental agents such as pollen or pollutants, that induce an esophagus-specific allergic response [5]. Concurrently, there is growing acknowledgment that immune mechanisms associated with food are pivotal in the pathogenesis of EoE. This is underscored by the significant overlap between EoE and IgE-mediated food allergy, common food triggers, and emerging ideas such as food-induced immediate esophageal responses and the role of pathogenic effector TH2 cells. Recent evidence supports that food-driven inflammation may occur via both IgE-dependent and IgE-independent pathways, highlighting the necessity for a comprehensive understanding of disease mechanisms. In this context, non-invasive and minimally invasive biomarkers serve as a valuable complement to traditional allergologic methods by providing additional tools to assess systemic or local immune activation linked to food-triggered diseases [6]. Esophageal epithelial cells contribute by producing chemotactic factors like TNF-α and eotaxin-3. Eosinophils, in turn, secrete TSLP and other pro-inflammatory mediators that promote tissue damage, fibrosis, and esophageal dysfunction. In Western populations, the estimated prevalence ranges from 40 to 150 cases per 100,000 individuals, affecting both children and adults, with a male-to-female ratio of approximately 3:1 [7].

EoE clinical manifestations are related to esophageal dysfunction and may include dysphagia, food impaction, heartburn, regurgitation, and the refusal to eat. To-date, EoE diagnosis relies on the histological analysis of esophageal mucosal biopsies obtained through upper gastro-intestinal endoscopy. The diagnosis is based on the identification of at least 15 eosinophils per high-power field (eos/HPF), along with typical symptoms and the exclusion of other causes of esophageal eosinophilia, as outlined in the most recent ESPGHAN guidelines [8,9].

As a consequence, the diagnosis and follow-up of EoE currently involve invasive and expensive procedures and are poorly tolerated, particularly by pediatric patients [9]. In pediatric settings, these limitations are further amplified by the need for general anesthesia during endoscopic procedures, increasing both clinical risk and healthcare costs.

To address these issues, in recent years, scientific research has focused on identifying non-invasive biomarkers that could facilitate earlier diagnosis, risk stratification, and disease monitoring. EoE pathogenesis involves infiltration by eosinophils and other immune cells (e.g., lymphocytes, mast cells, and dendritic cells), along with increased expression of inflammatory cytokines such as IL-4, IL-5, IL-9, and IL-13 [10].

Emerging diagnostic strategies under investigation include the analysis of salivary microRNAs (miRNAs) and plasma-derived extracellular vesicles (pEVs), both of which have shown encouraging levels of diagnostic sensitivity and specificity in preliminary studies [11]. Moreover, Venkateshaiah et al. (2021) [12] have focused on some very promising blood mRNAs. Nevertheless, several issues such as the lack of methodological standardization, heterogeneous findings, and the need for validation in large, multicenter cohorts are currently major barriers to clinical implementation [13].

This review aims to provide a critical overview of the current landscape of non-invasive biomarkers for EoE, with particular attention to their underlying pathophysiological mechanisms, current evidence of diagnostic accuracy, and potential applications in clinical practice. The goal is to offer a comprehensive and updated perspective that can guide future research and support clinical decision-making, promoting a more personalized and less invasive management of this complex disease.

## 2. Histological Analysis and Diagnostic Criteria

**Figure 1:**
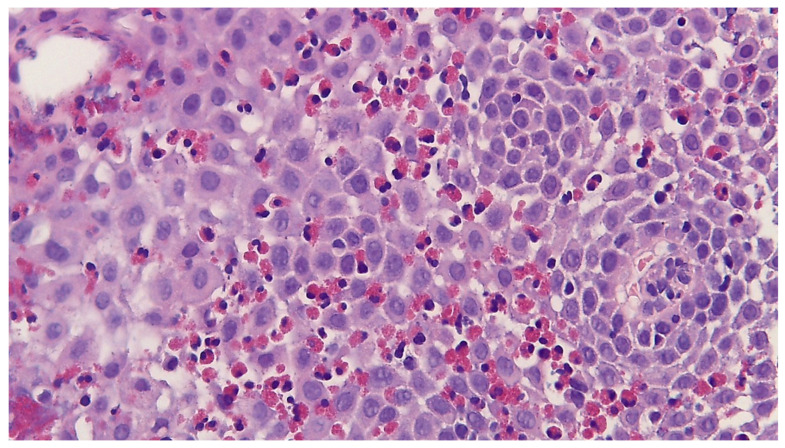
High intraepithelial eosinophilic count in a patient with EoE (hematoxilin–eosin staining, original magnification 400×).

According to the 2024 ESPGHAN guidelines [8], esophageal biopsy remains the cornerstone for the diagnosis of eosinophilic esophagitis (EoE). The diagnosis should be considered in the presence of symptoms of esophageal dysfunction unresponsive to proton pump inhibitor (PPI) therapy. On histology, EoE exhibits characteristic but not pathognomonic microscopic features, which can be further divided into major and minor ones [14]. Major features are considered necessary to diagnosis and include increased intraepithelial eosinophils (>15/HPF), eosinophil microabscesses, surface layering of eosinophils, alteration of superficial epithelial cells, and eosinophil degranulation, as seen in Figure 1. Instead, the identification of minor features is not required for diagnosis: marked basal zone hyperplasia, lengthening of the lamina propria papillae, increased lamina propria fibrosis and chronic inflammation, increased intercellular edema, and increased intraepithelial lymphocytes and mast cells belong to this category. Most of these features can explain the persistence of symptoms even after the normalization of intraepithelial eosinophils during treatment. Collins [15] and colleagues, aiming to establish a link between histological features and clinical course, as well as to improve the diagnosis and management of EoE, introduced the Eosinophilic Esophagitis Histology Scoring System (EoEHSS) [15]. To-date, however, it probably does not represent a conceptually significant, reliable, and reproducible tool [16]. Finally, the distribution of disease along the esophagus is quite variable as it may be patchy or focal, so the diagnosis strongly correlates with the number, site, and size of samples [15].

## 3. Minimally Invasive Biomarkers

Despite advances in understanding the pathogenesis of EoE, early diagnosis remains a clinical challenge, particularly in pediatrics, where it is often only recognized at an advanced stage with stenotic complications [9]. This diagnostic delay stems from the lack of early non-invasive biomarkers, forcing repeated endoscopic biopsies, invasive procedures especially in children, which require sedation [8]. Indeed, in the case of children with suspected EoE, the absence of early biomarkers delays diagnosis by an average of 2.3 years [17]. Prospective studies show that 68% of children visit the doctor urgently due to acute complications [7] with direct costs 3.5 times higher than that of early diagnosis [18]. In this context, during the last years, a growing body of research is exploring blood-, urine-, saliva- and stool-based biomarkers with the aim of complementing endoscopy-guided histology in EoE. The candidate biomarkers listed below in Table 1 fall into several mechanistic classes. Chemokines such as eotaxin-3 (CCL26) reflect the upstream signals that recruit eosinophils to the oesophageal mucosa. Eosinophil granule proteins (e.g., ECP, MBP, and EDN) and related cytoplasmic proteins (Galectin-10) mirror the activation and degranulation status of tissue-infiltrating eosinophils, while the oxidative by-product 3-bromotyrosine captures eosinophil-derived reactive species systemically. A broader inflammatory milieu is indexed by cytokines (IL-4, IL-5, IL-13, IL-15, and IL-33) and by immunoglobulin signatures such as total or food-specific IgG4. Metabolic mediators including 15(S)-HETE add an eicosanoid perspective. Beyond proteins biomarkers, circulating and extracellular-vesicle-associated microRNAs (e.g., miR-146a, miR-223, miR-21-5p, and miR-10b-5p) have shown dynamic changes with disease activity and corticosteroid therapy, whereas salivary miRNAs offer a completely non-invasive sampling matrix. Finally, an integrated immune-checkpoint panel based on the analysis of chemokine-receptor and T-cell markers demonstrates promise for differentiating EoE from gastro-oesophageal reflux disease and for monitoring treatment response. Here, we present a detailed analysis of recently found non-invasive biomarkers associated with EoE. For clearer classification, we subdivided them into two groups:

1. Blood-based biomarkers, mainly detectable in serum or plasma;
2. Non-blood-derived biomarkers, including those found in saliva, urine, stool, or other bodily fluids.

## 4. Minimally Invasive Biomarkers in Blood

### 4.1. Eosinophil-Associated Biomarkers

#### 4.1.1. Eotaxin-3 (CCL26)

Eotaxin-3, (CCL26; C-C Motif Chemokine Ligand 26), is a key chemokine for eosinophil chemotaxis and a potent activator of eosinophils, potentially triggering allergic airway inflammation. The CCL26 gene which encodes it has been found to be among the most highly induced in esophageal epithelium and peripheral blood during active inflammation in patients with EoE [19]. Pulmonary and vascular epithelial cells following stimulation with inflammatory interleukins, IL-4 and IL-13, produce chemokines, including eotaxin-3, activating CCR3 receptors, and leading to calcium mobilization and the recruitment of eosinophils and basophils from peripheral blood to sites of inflammation [40]. Furthermore, a study by Lin F. et al. suggest the role of eotaxin-3 in promoting epithelial–mesenchymal transition and even tumor growth and invasion [41].

Recent studies have highlighted the role of this chemokine in the pathogenesis of EoE, demonstrating a correlation between its serum levels and the histological activity of the disease. The one conducted by Blanchard et al. (2006) [19] was among the first to identify CCL26 as a possible biomarker in EoE patients because its levels correlated with disease activity. Subsequently, Jimenez-Aponte et al. (2014) [20], in a case–control study, observed that patients with EoE had statistically significantly higher serum levels of Eotaxin-3, as assessed by Quantikine Human Eotaxin-3 Immunoassay, than the healthy group, but that the concentration of Eotaxin-3 was not directly correlated with the eosinophil count in biopsies. On the contrary, Rabinowitz et al. (2022) [21] recently measured the levels of several hypothetical serological markers with Luminex assays, obtaining poor results. Indeed, they evaluated 11 analytes including IL-5, ll13, periostin, eotaxin-3, thymic stromal lymphopoietin, and immunoglobulins in a pediatric cohort composed of active EoE (*n* = 30), EoE in remission (*n* = 13), and controls (*n* = 34) and none were elevated or depressed compared to the different groups, nor were they correlated with peak esophageal eosinophilia, endoscopic features of EoE defined quantitatively by a validated EoE endoscopic reference score (EREFS) or esophageal thickness determined by endoscopic ultrasound.

Thus, CCL26 could be useful for monitoring response to therapy or identifying patients at risk of relapses and be a potential therapeutic target, since blocking CCL26 binding to its receptor CCR3 could be a future strategy to reduce eosinophil recruitment. However, there are obvious limitations in the standardization of measurement and individual variability in baseline levels; therefore, further studies are needed to validate its routine use.

#### 4.1.2. Eosinophil Cationic Protein (ECP)

Eosinophil cationic protein (ECP) is a protein released by activated eosinophils, following binding to the allergen that is taken up by immunoglobulin E (IgE) bound to the surfaces of eosinophils. On this basis, total serum IgE was evaluated in patients with EoE, and an increase was found [42]. Consequently, the diagnostic role of proteins excreted by eosinophils into peripheral blood has been studied. In a study by Cengiz (2019) [22], it was found that the level of eosinophilic cationic proteins was significantly higher in patients with EoE compared to healthy volunteers (20.4 vs. 8.8, *p* < 0.0001). The ROC curve was used to evaluate the predictivity of ECP for EoE, and it was found that with the ECP cut-off value of 13.9 μg/mL, the sensitivity was 80% and the specificity was 92.8%. The correlation of ECP with esophageal tissue eosinophil count, EREFS, and EoE symptoms was also analyzed, and it was found that only EREFS (*p* < 0.0001) and the presence of food impaction (*p* = 0.04) were significant.

However, data on the diagnostic accuracy of ECP for EoE are extremely rare. Although it is elevated in the serum of patients with active EoE, indicating eosinophil degranulation, it is also elevated in other allergic conditions, so the diagnostic data should be evaluated together with other parameters.

#### 4.1.3. Major Basophil Protein (MBP)

Major basic protein (MBP) is the predominant constituent of eosinophil granules. MBP is a 13.8 kDa basic protein produced as a larger inactive protein (proMBP). Its active form is stored in eosinophil granules. MBP is thought to induce direct tissue damage and also have indirect cytotoxic action by activating other inflammatory pathways. In patients with EoE, tissue eosinophils can degranulate, depositing MBP in the tissue. In a study by Peterson, et al. (2019) [24] eosinophil granule major basic protein 1 deposition, particularly eMBP1, was increased in esophageal biopsy specimens from symptomatic patients with EoE and may be a marker of disease activity. Furthermore, since eosinophils may lose their morphological identity when they degranulate, the measurement of released proteins such as MBP may better reflect disease activity than simply counting eosinophils. However, the assessment of eMBP1 described in the source was limited to the analysis of esophageal tissues by immunostaining and did not involve the measurement of MBP1 levels in blood.

MBP has also been investigated in serum, but with conflicting results. Dellon et al. (2015) [43] did not find statistically significant values between serum MBP levels between patients and controls at diagnosis and post-treatment. In contrast, in a posthumous study, Wechsler et al. (2021) [25] showed that plasma MBP-1 levels are predictive of maximum esophageal eosinophil count (PEC). Furthermore, in patients with EoE who responded to treatment (decrease in PEC to <15 eos/hpf), a significant reduction in plasma MBP-1 levels was observed after treatment compared to non-responders. This study is the first to demonstrate the utility of plasma MBP-1 as a biomarker for EoE. The authors also highlight the innovative methodological approach used for MBP-1 analysis, which included sample reduction and alkylation prior to ELISA to maximize recovery and detection. Thus, MBP is a promising serological biomarker for EoE, but protocols should be standardized to validate the results.

#### 4.1.4. Eosinophil-Derived Neurotoxin (EDN/EPX)

Eosinophil-derived neurotoxin (EDN, also known as Eosinophil protein X-EPX-) is a protein, a member of the RNase family, released by eosinophils. EDN is a neurotoxic protein of approximately 18 kDa with 89% homology to ECP (eosinophil cationic protein) and is involved in eosinophil inflammation as a product of the degranulation. Moreover, it has a role in the activation of Toll-like receptor 2 pathways. EDN is recognized as a key eosinophil-derived protein and a promising investigative biomarker for EoE. It has been studied in mucosa (luminal secretions—by esophageal string test and brushing— and tissue) and potentially in serum and feces, with several studies demonstrating its utility for diagnosis, the monitoring of disease activity (correlation with histology), and potential prediction of response to PPIs. As demonstrated by Min et al. 2017 [26], EDN has a good correlation as a serological biomarker in EoE, compared with controls. Moreover, Wechsler et al. 2021 [25] advanced the knowledge of EDN as a crucial potential combined biomarker in the diagnosis of EoE, both with invasive and non-invasive biomarkers. In the end, proteomic analysis suggests that EDN/RNase3 accumulated in esophageal tissue reflects the presence of mature eosinophils and correlates well with eosinophil count, although a corresponding variation at the mRNA level is not always observed [44].

#### 4.1.5. Galectin-10

Galectin-10, or Charcot–Leyden crystal protein (CLC), is a key protein in eosinophils and has an important role in the regulation of immune response by interacting with the surface of cationic ribonucleases in the eosinophil granules. Nevertheless, its association with EoE is poorly investigated, though Wechsler et al. 2021 [25] found a great correlation between cases and controls, especially when combined with other biomarkers. Indeed, studies on Gal-10 as a biomarker for EoE in serum and mucosa for diagnosis, treatment activities, and prediction of endoscopic severity could be further investigated.

### 4.2. Non-Eosinophil-Associated Biomarkers

#### 4.2.1. Interleukins (IL-15 and IL-4, IL-5, and IL-33, IL-13)

EoE, like other T-helper cell type 2 (Th2) diseases, employs an inflammatory mechanism, which includes interleukins among its mediators. There are several studies that report evaluated serum interleukins levels in EoE patients compared to control groups; however, there is currently no strong evidence for the use of these in combination or individually as biomarkers for EoE. Below, the evidence on the main interleukins studied for future diagnostic use is reported.

Venkateshaiah et al. (2020) [29] found that interleukin 15 (IL-15) mRNA levels correlate with esophageal eosinophilia in human EoE and are reduced in patients with improved EoE. Thus, IL-15 levels could reflect disease activity. However, the evaluation of this interleukin was performed at the tissue level and not in serum. Furthermore, in the results, it is observed that mice with overexpression of IL-15 have induced basal levels of interleukin 14 (IL-4) in blood and the esophagus. This suggests that IL-15 may influence serum IL-4 production in vivo. However, this study does not focus on the role of IL-4 as a direct biomarker for EoE, but rather on its link to IgE-mediated EoE pathogenesis, potentially influenced by IL-15. Ishihara et al. (2017) [30] analyzed the levels of various proteins, including some previously reported as potential biomarkers for eosinophilic esophagitis, such as interleukin 5 (IL-5) and interleukin 33 (IL-33); however, they were not significantly elevated in EoE patients.

Additionally, Sarbinowska et al. (2021) [28] measured serum levels of IL-5 and interleukin 13 (IL-13) in patients with dysphagia, including those diagnosed with EoE, and in a control group to evaluate their utility in diagnosing and monitoring the disease. At the time of diagnosis, there were no statistically significant differences between the two groups. However, statistically significant data emerged regarding IL-13 levels in relation to the treatment of the EoE group with proton pump inhibitors (PPIs): after 8 weeks of treatment, a significant increase in IL-13 was recorded (*p* = 0.03), and regarding endoscopic advancement assessed by EREFS, a strong and statistically significant negative correlation was found with fibrostenotic EREFS after treatment, and a moderate negative correlation with total EREFS after treatment. Despite their importance in the pathogenesis of EoE (such as the stimulation of eosinophil influx), serum interleukins alone do not appear to be reliable diagnostic or prognostic biomarkers for EoE. However, the negative correlation of IL-13 with fibrostenotic EREFS after treatment may warrant further investigation in the context of disease progression and therapeutic response.

#### 4.2.2. IgG4

IgG4 are a subclass of antibodies with low inflammatory affinity, involved in immune tolerance and chronic responses to allergens. Their serum levels may be useful in clinical diagnostics, although they are not specific. For this reason, they could be ideal candidates—when combined with other serum markers—for the non-invasive diagnosis of eosinophilic esophagitis (EoE). IgG4 are often found in the esophageal tissues of patients with EoE and have been correlated with histological severity [45]. Several studies have demonstrated that EoE in adults is associated with IgG4, where elevated IgG4 levels are more commonly found in EoE patients compared to controls [46], although this has typically been observed in esophageal mucosa samples obtained through biopsies or less invasive methods such as the Cytosponge or string test [31]. Other studies have also explored the relationship between circulating serum IgG4 levels and EoE [8]. Moreover, it has been found that serum IgG4 concentrations are higher in EoE patients compared to those with GERD and significantly decrease following effective EoE treatment. This suggests that serum IgG4 may serve as a potential biomarker for treatment response [32]. Additionally, Peterson et al. (2020) [31] investigated IgG4 antibodies in esophageal secretions in response to trigger foods (identified through elimination and reintroduction diets) compared to non-trigger foods. Most trigger foods showed elevated levels of both IgA and IgG4 antibodies. For IgG4 responses specifically, 20 out of 21 trigger foods exhibited the highest response values. However, significant variability in IgG4 levels was observed both between and within patients [31]. Despite this, the precise role of IgG4 in the pathogenesis of EoE has not yet been clearly defined [4]. IgG4 might play a role, possibly by blocking the IgE–allergen interaction or as part of localized immune responses in the esophagus [8]. This is supported by some studies proposing that EoE is an IgG4-mediated disease, showing that EoE in adults is associated with IgG4 and not mediated by IgE [47]. Finally, although its exact role in pathogenesis is not fully understood, some studies link IgG4 to local immune responses and suggest a potential role in distinguishing EoE from GERD, as well as in monitoring treatment response through both serum and mucosal levels.

#### 4.2.3. 15(S)-HETE

15(S)-hydroxyeicosatetraenoic acid (15(S)-HETE) is a metabolite derived from the molecular pathway of ALOX-15, an enzyme involved in the pathogenesis of Th2-mediated diseases such as eosinophilic esophagitis (EoE). In particular, studies [46,48] conducted on biopsies from EoE patients demonstrated that ALOX-15 mRNA expression is significantly increased, showing 95% positivity in EoE cases compared to controls, in which expression was consistently negative. Therefore, a circulating metabolite derived from this enzyme, such as 15(S)-HETE, could be a promising candidate as a biomarker for EoE. In fact, Lu et al. (2018) [33] reported that 15(S)-HETE may play a role as a biomarker when used in combination with other selective metabolites for EoE, such as eosinophil-derived neurotoxin (EDN), eotaxin-3, and IL-1, both for diagnosis and disease management. However, when measured alone, 15(S)-HETE cannot serve as a selective marker for the disease, as it does not effectively distinguish EoE from other Th2-mediated conditions. In a study published in August 2024 [34], researchers investigated various biomarkers in the serum and saliva of children with EoE, including 15(S)-HETE. Serum levels of 15(S)-HETE were elevated in both children with active EoE and those in remission compared to healthy controls. In contrast, no significant differences in salivary levels were observed among the different groups. In this study as well, the researchers evaluated biomarker panels and found that a reduced panel of four markers—AEC (absolute eosinophil count), EDN, and specific IgE to egg white and wheat—could accurately distinguish children with active EoE from healthy individuals. As a result, 15(S)-HETE was not included in the final panel, since, unlike the other markers, its levels did not decrease in patients in remission.

#### 4.2.4. MicroRNAs in Plasma and Plasma-Derived Extracellular Vesicles (EVs)

MicroRNAs (miRNAs) are endogenous short RNA molecules of 19 to 25 nucleotides in length that regulate the expression of target genes by post-transcriptional silencing. The microRNA profile in the plasma of EoE patients was studied by Lu et al. in 2012 [35]. They identified miR-146a, miR-146b, and miR-223 as the most abundant circulating miRNAs in plasma differentially expressed between EoE and healthy individuals. Furthermore, they observed that miR-146a and miR-223 levels were reversed in patients who responded to glucocorticoid therapy.

A few years later, Sawant et al. (2015) [49] suggested the role of microRNA-21 (miR-21) as a potential biomarker for allergic inflammatory diseases in children, including eosinophilic esophagitis (EoE), as its levels were elevated in both esophageal tissues and in the serum of affected children. More recently, Tarallo et al. [36] suggested that MiR-21-5p and MiR-223, which have the most potential as markers for EoE non-invasive biomarker, also have a good correlation in disease monitoring both in adult and children. Unfortunately, no other studies on plasma and/or serum microRNAs have been performed.

Only recently have microRNAs (miRNAs) been studied in plasma-derived extracellular vesicles (EVs) (pEVs) from patients with active disease, after treatment, and in the control cohort. A difference in the miRNA load of pEVs was observed between the different conditions studied. Compared to controls, upregulated (miR-10b-5p and miR-125a-5p) and downregulated (miR-224-5p, miR-221-3p, let-7d-5p, and miR-191-5p) miRNAs were identified in active EoE; however, the best combination to distinguish the two groups is the combination of miR-221-3p and miR-10b-5p [37]. Comparing EoE samples before and after treatment, miR-30a-3p was found to be a potential biomarker for treatment monitoring. However, further studies exploring the role of EVs in EoE are needed.

#### 4.2.5. mRNA from Blood

Circulating mRNAs in blood are widely used as molecular biomarkers for the diagnosis, monitoring, and treatment of various diseases. In general, omics analyses, such as genomics (comprehensive analysis of DNA) and transcriptomics (comprehensive analysis of RNA transcripts, particularly mRNA), can be performed using blood as a surrogate tissue. This strategy allows for the avoidance of invasive procedures such as tissue biopsies [50]. In this context, different mRNAs were studied as a precursor of inflammatory proteins, as interleukin, or their receptors. As shown by Venkateshaiah et al. (2021) [12] in their first work, blood mRNA levels of receptors of IL-15 responsive T-cell (Vα24, Jα18, γδ T, and αβ T) and IgE (FcεRI) are reduced in EoE compared to GERD and the correlation analysis of FcεRII, Jα18, and δTCR are the positive predictors that discriminate EoE from GERD. Moreover, in a second work [51], they propose mRNA levels of CD274, CD101, CXCR6, TCRδ, Jα18, and FCεRII as a novel non-invasive biomarker panel for monitoring the EoE disease in patients before and after treatment and to differentiate EoE disease from GERD in patients, emphasizing TCRδ, Jα18, and FCεRII, which seem to be really promising for monitoring and diagnosing EoE. In the end, other sets of mRNA were analyzed—PPL, JUP, SerpinB13, CES2, KLK13, SerpinB3, CRNN, KRT4, SerpinB4, CSTB, SerpinB5, CYSRT-1, LYPD3, SFN, FABP5, MAL, SPRR1A, Galectin 3, MUC22, and TGM3—without highlighting significant differences between cases and controls [52].

## 5. Minimally Invasive Biomarkers in Other Biological Fluids (Saliva, Mucus, and Urine)

### 5.1. MicroRNAs as Potential Salivary Biomarkers

MicroRNAs (miRNAs) are increasingly being studied in several allergic diseases, including EoE, but few have examined them in saliva from patients with EoE. Jhaveri et al. (2023) [38] studied the profile of salivary microRNAs in order to evaluate whether they could actually be useful in distinguishing children with EoE. The analysis was conducted on 150 samples from children undergoing esophagogastroduodenoscopy, of which, 50 showed histological alteration and 100 showed no pathological alteration. Of the 56 salivary miRNAs reliably identified, miR-205-5p showed the most significant difference between EoE and non-EoE groups (*p* value = 0.029). Additionally, six other microRNAs, including miR-26b-5p, miR-27b-3p, Let-7i-5p, miR-142-5p, miR-30a-5p, and miR-205-5p, were able to differentiate EoE samples with a sensitivity of 70% and a specificity of 68%.

Instead, Bhardwaj N. et al. (2020) [39] evaluates the role of MiR -4668 in characterizing EoE and monitoring therapy with swallowed topical corticosteroids. MiR-4668-5p has never been previously identified as a target in any allergic disease, and its levels were significantly higher in EoE patients compared to the control cohort; furthermore, its levels decreased in the majority of subjects treated with fluticasone. Thus, microRNAs are potential future non-invasive biomarkers that can distinguish between subjects with and without the disease and reflect response to treatment.

### 5.2. Eosinophil Cationic Protein (ECP) in Mucus Secretions

As previously mentioned, eosinophil cationic protein (ECP) is known to be upregulated in patients with eosinophilic esophagitis (EoE) compared to healthy individuals and patients with other esophageal conditions, such as gastroesophageal reflux disease (GERD). This upregulated ECP protein is present not only in tissue biopsies, but also in serum and potentially in esophageal mucus of patients with EoE. From this assumption, Souza et al. (2022) [23] established that there is a relationship between the presence and detection of eosinophilic cationic protein (ECP) in esophageal mucus and its potential use as an ancillary diagnostic tool for eosinophilic esophagitis (EoE). Instead of a direct quantitative measurement of ECP as would be performed with a standard biochemical test, the study developed a ligand peptide (E5) that specifically binds to ECP. The diagnostic measurement is then based on the reactivity of this peptide with patients’ esophageal mucus, which indirectly indicates the presence (and potentially the amount) of ECP. This test demonstrated a sensitivity of 84.62% and a specificity of 82.72% in distinguishing EoE. Furthermore, the results correlate positively with eosinophil counts in esophageal tissue. This approach offers the potential for a less invasive auxiliary diagnosis compared to endoscopic biopsies. It would be interesting to evaluate eosinophil cationic protein levels in matrices other than serum, such as saliva, to make diagnostic and monitoring tools for EoE increasingly less invasive.

### 5.3. Urine Sample: 3-Bromotyrosine

Urine is a relatively understudied sample type in the search for non-invasive biomarkers for eosinophilic esophagitis (EoE). In this context, Cunnion et al. (2016) [27] investigated 3-Bromotyrosine (3-BT). In this study, they used mass spectrometry with the EoQUIK assay (Eosinophil Quantitated Urine Kinetic, EoQUIK) as the detection technique. They compared 3-BT levels between cases and controls, normalizing the values to creatinine levels. This study was very promising in terms of the correlation between cases and controls. However, the analytical technique used for 3-BT detection is quite expensive and requires a specialized operator. For this reason, the authors of this review explored other inflammation-related biomarkers mediated by eosinophils, not directly associated with EoE, but which could potentially be implemented in future clinical research. Indeed, Lönnkvist et al. (2001) [53] analyzed non-invasive markers from blood, serum, and urine associated with eosinophilic (or eosinophil-related) diseases such as asthma for monitoring during treatment. Furthermore, Klonoff-Cohen & Polavarapu (2016) [54] reviewed the literature on the use of Eosinophil Protein X (EPX/EDN) as a marker for childhood asthma. Although the results reported were not predictive for pediatric asthma, these markers could be further investigated in other eosinophil-associated conditions, such as EoE.

## 6. Discussion

Eosinophilic esophagitis (EoE) is a chronic inflammatory disease of the esophagus, whose etiology is still unclear [8,9]. Despite a recent review highlighting various aspects of the pathogenesis of this disease [55,56] and different studies based on in vitro, cellular, and animal assays, a specific trigger factor has not yet been defined. Mucosal biopsies and eosinophil count are currently the gold standards for diagnosis and clinical follow-up, as reported by the latest ESPGHAN guidelines [8], despite esophagogastroscopy being a highly invasive test, especially in pediatric patients.

As discussed within the various sections of this review, the currently used and validated biomarkers for EoE diagnosis and monitoring have poor specificity for the disease. This feature makes it more complex to distinguish EoE from other clinical conditions with overlapping manifestations. For instance, it is difficult to differentiate EoE from other esophageal diseases that are associated with esophagitis and a concomitant increase in inflammatory mediators, both at the tissue and systemic level. Furthermore, as highlighted by the ESPGHAN guidelines [8], there is actually a limited correlation between the current diagnostic method (biopsies and eosinophil count) and the reported symptoms. Indeed, very often, symptom severity is not associated with the histological picture. In agreement with this latter consideration, ref. [32] recently stressed the need for the development of new diagnostic tools for both diagnosis and follow-up, which should preferably be non-invasive or less invasive, especially in the pediatric setting. In this context, we believe that in the near future, the search for circulating and less invasive biomarkers in biological fluids will have a significant impact on the clinical management of EoE [10]. However, despite the increased interest in these biomarkers, there are still a lot of limitations. Most of the evidence currently available is based on small, single-center cohorts, non-standardized sampling techniques, and different analytical approaches, all of which restrict cross-study comparability. Consequently, even though several biomarkers, including EDN/EPX, specific circulating miRNAs, and whole-blood mRNA panels, exhibit encouraging diagnostic or monitoring potential, their integration into standard clinical practice will necessitate strong multicenter validation and consistent methodological procedures.

In this review, we analyzed several articles focusing on the identification of possible blood serum markers, such as galectin-10 [25], eotaxin-3 [19,20,21], and eosinophil cationic protein (ECP) [22,23], in relation to eosinophil activity. Instead, interleukins such as IL-5 and IL-13 [28], IL-15 [29], IL-33 [30], and IgG4 [31,32] may also help us understand the immune system problems associated with EoE, although they are not specific to eosinophils. In addition to interleukins, circulating miRNAs have been considered as a new possible class of biomarkers for EoE. Circulating serum miRNAs, such as miR-146a [35], miR-223, and miR-21-5p [36], have shown promising results in EoE, being upregulated in EoE patients. In fact, although miR-223 and miR21-5p [36] have already been studied extensively in adults, these have also been found at higher levels in the mucosa and plasma of children affected by EoE, respectively. Furthermore, we believe that among the circulating miRNAs currently identified, particular attention should be paid to miR-223. Indeed, this miRNA has been found to be upregulated in relation to EoE onset, while it decreased following PPI treatment. Furthermore, unlike others that have been tested, and which also appear to be of interest, it is important to underline that this miRNA is expressed at the genetic level on the X chromosome. This peculiarity could be linked to the higher incidence of the disease in male patients compared to females (3:1) [57,58]; however, genes and miRNAs not linked to the X chromosome are under study [7,59].

In addition to blood biomarkers, as discussed previously, other biological fluids can be considered for the development of non-invasive biomarkers for diagnosis or follow-up in various pathologies [32]. Among the various biological fluids, much attention has recently been paid to saliva [60]. Salivary miRNAs are potential biomarkers that can be evaluated in clinical practice. They may also represent a non-invasive biomarker option, although further studies are needed before clinical use. In saliva, miR-205-5p had high expression and was particularly different between patients and controls in a pediatric study [38]; moreover, miR-4668-5p [39] was detectable in pediatric patients as it was upregulated compared to controls without the disease [37,38]. Unfortunately, not all diagnostic centers have the equipment to handle saliva samples consistently and there are no standardized procedures, as they are not routinely performed. Stool and urine tests can also provide useful information, but they are not used frequently due to the possibility of contamination with bacteria and the needs of specific management [25,26,27,53,54].

To-date, the diagnosis and follow-up of EoE still have several limitations. Although there is a growing interest, the lack of multicenter or “omics” studies prevents a comprehensive and truly informative picture of the disease. Furthermore, most available studies are limited by the small number of enrolled patients, as EoE is still considered a low-incidence disease. All these elements underscore the urgent need to expand our knowledge of this condition. Looking ahead, we believe that, due to the complexity of EoE, a single biomarker is unlikely to be adequate. Currently, no evaluated non-invasive biomarkers can replace endoscopy, but many can serve as additional tools to stratify risk, monitor treatment response, or reduce the frequency of invasive procedures when used within validated multi-assay panels. Thus, a panel of markers combining cytokines, miRNAs, and eosinophil-derived proteins could increase the accuracy of diagnosis, monitor the disease course and predict treatment efficacy. Repeated endoscopies could be reduced with a validated biomarker-based approach, which would be of paramount importance in pediatric patients. This change would allow for more dynamic monitoring, reduced healthcare burdens, and greater patient comfort [3,25,28]. Despite progress, current studies often suffer from small sample sizes, inconsistent methodologies, and outdated diagnostic criteria [43].

To move forward, multicenter studies are essential. Large-scale testing is needed to confirm the reliability of biomarkers in diverse populations [32]. Although there are still hurdles to overcome, a concerted effort that includes robust clinical validation and interdisciplinary collaboration could revolutionize the management of EoE by minimizing invasive procedures and improving therapeutic approaches. The development of non-invasive biomarkers is a crucial step towards precision medicine in EoE.

## Figures and Tables

**Table 1:** Overview of candidate minimally invasive biomarkers.

Category	Biomarker	Molecular Class	Biological Matrix	Potential Use	Results	References
Eosinophil-Associated	Eotaxin-3 (CCL26)	Chemokine-Protein	Serum	Diagnosis, Monitoring and Therapeutic Target	High levels in EoE, but variable correlation with eosinophil count. Potential for monitoring therapy.	Blanchard et al., 2006 [19]; Jimenez-Aponte et al., 2014 [20]; Rabinowitz et al., 2022 [21].
	Eosinophil Cationic Protein (ECP)	Eosinophil Granule Protein	Serum, Esophageal Mucus	Diagnosis, Correlation with Endoscopic Severity.	Sensitivity 80%, specificity 92.8% at cut-off 13.9 μg/mL. Correlates with EREFS and dietary impact.	Cengiz, 2019 [22]; Souza et al., 2022 [23].
	Major Basic Protein (MBP)	Eosinophil Granule Protein	Esophageal Tissue, Serum	Monitoring	Elevated in tissue, but conflicting results in serum. Potential for monitoring therapy response.	Peterson et al., 2019 [24]; Wechsler et al., 2021 [25].
	Eosinophil-Derived Neurotoxin (EDN/EPX)	Eosinophil RNAse-Protein	Serum, Feces, Esophageal Mucus	Diagnosis, Monitoring and Prediction of Response to PPI Drugs	EDN concentrations distinguish active EoE from remission and correlate with peak eosinophil counts; included in validated multi-analyte panels for non-invasive monitoring.	Min et al., 2017 [26]; Wechsler et al., 2021 [25].
	Galectin-10 (CLC)	Eosinophilic Cytoplasmatic Protein	Serum	Diagnosis (in Combination with Other Biomarkers).	Elevated Gal-10, particularly when combined with EDN and ECP, enhances diagnostic accuracy; adult data are presently scarce.	Wechsler et al., 2021 [25].
	3-Bromotyrosine (3-BT)	Brominated type amino acid chemical compounds	Urine	Diagnosis and Monitoring	Promising but detection techniques are expensive.	Cunnion et al., 2016 [27].
Non-Eosinophil-Associated	Interleukine (IL-4, IL-5, IL-13, IL-15, IL-33)	Cytokine-protein	Serum	Pathogenetic Role, Monitoring Therapy	Circulating Th2 cytokines show inconsistent elevation; IL-13 rose after PPI therapy but lacked correlation with eosinophil density, limiting their utility as stand-alone biomarkers.	Sarbinowska et al., 2021 [28]; Venkateshaiah et al., 2020 [29]; Ishihara et al., 2017 [30].
	Total or Specific IgG4	Antibodies-proteine	Serum	Differential Diagnosis (vs. GERD), Monitoring Response to Therapy	IgG4 levels exceed those in GERD and decline with effective therapy, supporting a role in treatment monitoring, yet high inter-individual variability precludes diagnostic use.	Peterson et al., 2020 [31]; Visaggi et al., 2023 [32].
	15(S)-hydroxyeicosatetraenoic acid (15(S)-HETE)	Polyunsatured fatty acid	Serum	Diagnosis (in Combination with Other Biomarkers).	Elevated in active EoE and remission, but not included in diagnostic panels due to lack of specificity.	Lu et al., 2018 [33]; Thulin et al., 2024 [34].
Circulating miRNAs	miR-146a, miR-223, miR-21-5p	microRNA	Plasma	Diagnosis, Monitoring Response to Glucocorticoids	miR-146a and miR-223 reversible post-therapy. miR-21-5p elevated in EoE.	Lu et al., 2012 [35]; Tarallo et al., 2025 [36].
	miR-10b-5p, miR-221-3p		Extracellular Vescicles (pEVs)	Diagnosis	Differences between active EoE and controls	Grueso-Navarro et al., 2025 [37].
	miR-205-5p, miR-4668-5p		Saliva	Diagnosis, Monitoring Therapy	miR-205-5p differentiates EoE vs. controls. miR-4668-5p reduced post corticosteroid therapy.	Jhaveri et al., 2023 [38]; Bhardwaj et al., 2020 [39].
Whole-Blood mRNA Panel	CD274, CD101, CXCR6, TCRδ, Jα18, FCεRII	mRNA	Whole Blood	Differential Diagnosis (vs. GERD), Monitoring Response to Therapy	Promising panel to discriminate EoE from GERD and monitor activity.	Venkateshaiah et al., 2021 [12]

## Data Availability

No new data were created or analyzed in this study.

## Abbreviations

The following abbreviations are used in this manuscript:

EoE	Eosinophilic Esophagitis
Th2	T-Helper Type 2 (immune response)
Eos	Eosinophil
HPF	High-Power Field
PPI	Proton Pump Inhibitor
ESPGHAN	European Society for Pediatric Gastroenterology, Hepatology, and Nutrition
GERD	Gastroesophageal Reflux Disease
EoEHSS	Eosinophilic Esophagitis Histology Scoring System
CCL26	Chemokine (C–C motif) Ligand 26 (Eotaxin-3)
ECP	Eosinophil Cationic Protein
MBP	Major Basic Protein
EDN/EPX	Eosinophil-Derived Neurotoxin/Eosinophil Protein X
CLC	Charcot–Leyden Crystal Protein (Galectin-10)
3-BT	3-Bromotyrosine
IL	Interleukin
IL-4,	Interleukin 4
IL-5	Interleukin 5
IL-13	Interleukin 13
IL-15	Interleukin 15
IL-33	Interleukin 33
IgG4	Immunoglobulin G4
IgE	Immunoglobulin E
15(S)-HETE	15(S)-Hydroxyeicosatetraenoic Acid
miRNA/miR	MicroRNA
pEVs	Plasma-Derived Extracellular Vesicles
EVs	Extracellular Vesicles
mRNA	Messenger RNA
DNA	Deoxyribonucleic Acid
RNA	Ribonucleic Acid
TCRδ/TCR	T-Cell Receptor Delta/T-Cell Receptor
Jα18	Joining Alpha Segment 18 (T-cell receptor gene segment)
CD274	Cluster of Differentiation Markers 274
CD101	Cluster of Differentiation Markers 101
CXCR6	C-X-C Chemokine Receptor 6
FCεRII/FCεRI	Low-Affinity/High-Affinity IgE Receptor
AEC	Absolute Eosinophil Count
TNF-α	Tumor Necrosis Factor alpha
TSLP	Thymic Stromal Lymphopoietin
EREFS	Endoscopic Reference Score for Eosinophilic Esophagitis
